# Predictive error detection in pianists: a combined ERP and motion capture study

**DOI:** 10.3389/fnhum.2013.00587

**Published:** 2013-09-26

**Authors:** Clemens Maidhof, Anni Pitkäniemi, Mari Tervaniemi

**Affiliations:** ^1^Cognitive Brain Research Unit, Cognitive Science, Institute of Behavioural Sciences, University of HelsinkiHelsinki, Finland; ^2^Department of Music, Finnish Centre of Excellence in Interdisciplinary Music Research, University of JyväskyläJyväskylä, Finland; ^3^Department of Psychology, University of JyväskyläJyväskylä, Finland

**Keywords:** EEG, performance monitoring, music performance, motor control, musical expertise

## Abstract

Performing a piece of music involves the interplay of several cognitive and motor processes and requires extensive training to achieve a high skill level. However, even professional musicians commit errors occasionally. Previous event-related potential (ERP) studies have investigated the neurophysiological correlates of pitch errors during piano performance, and reported pre-error negativity already occurring approximately 70–100 ms before the error had been committed and audible. It was assumed that this pre-error negativity reflects predictive control processes that compare predicted consequences with actual consequences of one's own actions. However, in previous investigations, correct and incorrect pitch events were confounded by their different tempi. In addition, no data about the underlying movements were available. In the present study, we exploratively recorded the ERPs and 3D movement data of pianists' fingers simultaneously while they performed fingering exercises from memory. Results showed a pre-error negativity for incorrect keystrokes when both correct and incorrect keystrokes were performed with comparable tempi. Interestingly, even correct notes immediately preceding erroneous keystrokes elicited a very similar negativity. In addition, we explored the possibility of computing ERPs time-locked to a kinematic landmark in the finger motion trajectories defined by when a finger makes initial contact with the key surface, that is, at the onset of tactile feedback. Results suggest that incorrect notes elicited a small difference after the onset of tactile feedback, whereas correct notes preceding incorrect ones elicited negativity before the onset of tactile feedback. The results tentatively suggest that tactile feedback plays an important role in error-monitoring during piano performance, because the comparison between predicted and actual sensory (tactile) feedback may provide the information necessary for the detection of an upcoming error.

## Introduction

Performing a piece of music is a highly demanding task, involving several cognitive and motor processes (for reviews, see e.g., Palmer, 1997; Münte et al., 2002; Zatorre et al., 2007). Although professional musicians spend thousands of hours of deliberate practice to master their instrument (Ericsson et al., 1993; Ericsson and Lehmann, 1996; Sloboda et al., 1996), they make errors occasionally. To detect errors, which represent deviations from intended goals and actions and their effects, humans have to constantly monitor their ongoing behavior and its outcomes. The present study aimed at investigating the neurophysiological correlates of error-related processes during music performance. To relate neurophysiological findings to different movement stages, we used a new exploratory paradigm in which EEG and 3D movement data with a motion capture system were concurrently recorded.

Most previous neuroscientific research has focused on errors committed during various choice-reaction time tasks (for reviews, see Nieuwenhuis et al., 2004; Yeung et al., 2004; van Veen and Carter, 2006; Taylor et al., 2007). A prominent finding was a sharp negative deflection in the event-related potential (ERP) peaking around 50–100 ms after an incorrect response, termed the error-related negativity or error negativity (ERN and Ne, respectively; Falkenstein et al., 1990; Gehring et al., 1993). The ERN can be elicited independently of the modality in which the stimulus is presented (Falkenstein et al., 2000), and independently of the effector (hand or foot) with which the incorrect response is made (Holroyd et al., 1998). Evidence from EEG source localization studies, functional neuroimaging studies, as well as from single-unit recordings from primates indicate that the ERN receives major contributions from the dorsal part of the anterior cingulate cortex (dACC; for a review, see Ridderinkhof et al., 2004).

Several theories have been put forward with regard to the functional role of the ERN. According to the comparator theory, the ERN reflects the outcome of a process that compares the neural representation of the actual response with the correct response (Falkenstein et al., 2000). By contrast, the conflict monitoring theory (Carter, 1998; Botvinick et al., 2001; van Veen et al., 2001) assumes that the dACC monitors for cognitive conflict occurring when two competing response representations are activated (as for example with the Stroop effect). Within this framework, errors are a special case of high conflict, and the ERN is elicited when the representation of an incorrect response crosses a threshold so that an actual response is being made. The reinforcement learning theory of the ERN (Holroyd and Coles, 2002; Nieuwenhuis et al., 2004), which can be viewed as an extension of the comparator theory, posits that the ERN is elicited when the outcome of an event is worse than expected. In that view, the basal ganglia monitor ongoing events and predict whether they will be better or worse than expected. If an event is predicted to be worse than expected, the basal ganglia signal this with a phasic decrease in dopaminergic activity in the ACC, which gives rise to the ERN. However, there is an ongoing debate as to the degree to which each theory can account for the existing findings.

Another important finding in the domain of performance monitoring was the feedback ERN (for a review, see Nieuwenhuis et al., 2004). This component is elicited around 250 ms after negative performance feedback indicating loss or punishment in time-estimation, guessing, and gambling tasks (e.g., Miltner et al., 1997; Hajcak et al., 2005, 2007), and is presumably generated in the ACC (Nieuwenhuis et al., 2004). However, there is also evidence that the feedback ERN is elicited not only by negative feedback, but also by unexpected positive feedback (Oliveira et al., 2007; Ferdinand et al., 2012), indicating that the medial frontal cortex is sensitive to violations of expectancy in general, regardless of the specific valence of an event.

The processing of errors and unexpected feedback has also been investigated during speech production and musical performance. In the speech domain, vocal errors committed during, for example, the Stroop color-word task (Masaki et al., 2001), a picture naming task (Riès et al., 2011), or during the monitoring of internal speech in Go/No-go tasks (Ganushchak and Schiller, 2006, 2008) elicited a negative potential shortly after the onset of an incorrect response that highly resembled the ERN observed in non-linguistic tasks. This indicated that the ERN reflects more domain-general response monitoring functions.

In addition to the monitoring of internal speech, auditory feedback provides an important source of information about ongoing speech acts, and can be used to control vocal fundamental frequency. Studies investigating the processing of manipulated auditory feedback (i.e., pitch- or time-shifted feedback) during vocalizations in humans and non-human primates reported that the motor-induced suppression of auditory cortical responses (i.e., the inhibitory effects within the auditory cortex during vocalization, as compared to listening) is decreased during feedback perturbations (e.g., Houde et al., 2002; Heinks-Maldonado et al., 2006; Eliades and Wang, 2008; Behroozmand and Larson, 2011; Behroozmand et al., 2011). It has been argued that the underlying mechanisms are based on internal forward models in the auditory domain, and that the dampening of sensory input can help to differentiate self-produced from externally-generated sounds. In that view, an internal forward model receives information about the ongoing motor command in the form of an efference copy. The forward model can predict the sensory consequences of an action by integrating information about the current state of the system and this efference copy (Wolpert et al., 1995; Desmurget and Grafton, 2000). If the comparison between actual (in the form of reafferent sensory and tactile/proprioceptive information) and predicted feedback yields a match, the resulting small prediction error leads to minimal responses in the auditory cortex. In the event of a mismatch between the predicted and actual consequences, an error signal is generated that can be used to cancel the inhibitory effects within the auditory cortex. Furthermore, it is assumed that the error signal can be used for rapid adjustments of ongoing motor activity.

In the music domain, the processing of occasionally manipulated auditory feedback (compared to normal feedback) during piano performances elicited an increased N100 response and a negative potential around 200 ms that was interpreted as a feedback ERN (Katahira et al., 2008; Maidhof et al., 2010). The feedback ERN elicited by unexpected pitch shifts of the feedback showed larger amplitudes during performance than during listening (Maidhof et al., 2010), resembling the results in the speech domain. This indicates that manipulated notes were more unexpected when they were self-generated, which has been explained—in a similar manner to the findings in the speech domain—in terms of internal forward models (Katahira et al., 2008; Maidhof et al., 2010).

Furthermore, several recent studies investigated rare pitch errors (i.e., playing an incorrect note on the keyboard) in highly-skilled pianists performing pieces of music and fingering exercises from memory. Results showed that pitch errors, compared to correct notes, elicited a negative component in the ERP that already peaked approximately 70–100 ms prior to the onset of errors, and thus prior to the auditory feedback of the wrong note (termed “pre-error negativity” or “preERN,” Herrojo Ruiz et al., 2009; Maidhof et al., 2009; Strübing et al., 2012). The negative component showed a frontocentral scalp topography (Herrojo Ruiz et al., 2009), regardless of whether errors were committed with the right or left hand (Maidhof et al., 2009). Furthermore, it was elicited even in the complete absence of auditory feedback (Herrojo Ruiz et al., 2009), can be altered in pianists with focal dystonia (Strübing et al., 2012), and is presumed to be generated in the ACC (Herrojo Ruiz et al., 2009).

On the behavioral level, these studies reported that erroneous keys were pressed with a lower velocity than correct keys, which resulted in decreased intensities of wrong notes (Herrojo Ruiz et al., 2009; Maidhof et al., 2009; Herrojo Ruiz et al., 2011; Strübing et al., 2012). In addition, errors and the following notes were executed slower, that is, the inter-onset intervals (IOIs) from (a) the preceding note to the incorrect note (calculated as *n* minus *n* − 1); and (b) from the incorrect note to the subsequent correct note (calculated as *n* + 1 minus *n*) were prolonged. Interestingly, a recent study also showed that correct notes immediately preceding wrong keystrokes (“pre-error notes”) were pressed with decreased velocities, although not to the same degree as errors (Palmer et al., 2012). This latter observation is consistent with the notion that errors can influence surrounding events in a sequence, such that pre-error notes “inherit” some features of the following error (decreased intensity), but are still correct with regards to the pitch property.

The ERP effect prior to pitch errors during piano performance (Herrojo Ruiz et al., 2009, 2011; Maidhof et al., 2009; Strübing et al., 2012) has been hypothesized to reflect error detection processes, which do not rely on auditory feedback and could be based on internal forward mechanisms. However, the precise movement stages during which the pre-error negativity occurs have remained unclear, as has the role played by tactile feedback during musical performance for error-related processes.

The aim of the present study is to address the following issues:

1. In previous studies, the direct comparison of correct and incorrect notes was confounded by their different IOIs. Because of the possibility that ERPs of wrong notes (longer IOIs before the incorrect note) and the previous correct notes (shorter IOIs) overlap, the different IOIs could possibly have influenced the ERP effect prior to the erroneous notes. In the present study, we therefore compared incorrect and correct keystrokes that were executed at comparable tempi. If the ERP effect before errors reflects mainly error-related processes and not tempo differences, it should be elicited even when correct and incorrect notes show a similar tempo. IOIs were always calculated as *n* minus *n*-1, thus IOIs refer to the pre-note intervals.
2. Previous studies related the electrophysiological data to the time-point when the key was almost fully pressed (i.e., the point at which the MIDI [Musical Instrument Digital Interface] signal is generated by a digital piano upon depression of a key). In the present study, we exploratively recorded 3D movement data of participants' fingers simultaneously with the EEG to investigate the underlying movements and the role of tactile feedback for error monitoring. Two previous studies (Goebl and Palmer, 2008; Palmer et al., 2009) showed that kinematic landmarks like acceleration peaks in the finger trajectories provide a measure of the available tactile information. Specifically, a finger-key landmark (FK landmark) can occur when a finger arrives at the piano key surface and changes its acceleration abruptly (before the key is actually pressed down), reflecting the onset of tactile feedback (see also Goebl and Palmer, 2009, 2013). In the present study, we analyzed the finger acceleration profiles of correct and incorrect keystrokes, and additionally computed the ERPs time-locked to the onsets of finger-key landmarks. We hypothesized that if a difference between the ERPs of correct and incorrect keystrokes occurs prior to FK landmarks, tactile information does not contribute to the pre-error negativity. In contrast, an ERP difference after FK landmarks would indicate that tactile feedback might play an important role for error-related processes during music performance.
3. Based on recent behavioral evidence showing that correct pre-error notes inherit some incorrect properties similar to errors (Palmer et al., 2012), we also investigated the ERPs to pre-error notes and compared them to other correct notes.

## Materials and methods

### Participants

Nineteen pianists participated in the study. Participants were current or former students at Finnish universities with professional music programs or music conservatories. Based on listening to the performances, one participant was excluded because the performance showed that the stimuli could not be produced fluently and included too many interruptions. Six participants were excluded because preliminary analyses indicated that their performances were too slow (mean IOI clearly above 200 ms). One participant was excluded because the markers were not correctly recorded by the motion capture system, and one participant was excluded because of excessive alpha activity. Thus, the data of 10 pianists (6 females; mean age: 23.7 years, *SD* = 5.5 years) were analyzed. They had, on average, 14.2 years of formal musical training (*SD* = 7.3 years), and had begun playing the piano between 4 and 10 years of age. On average, participants spent 2.7 h (*SD* = 1.2 h) daily on piano practice. Handedness was assessed with a revised version of the Edinburgh Handedness Inventory, in which three original activities were discarded (opening box, broom, drawing), and one new one was added (computer mouse; see http://homepage.ntlworld.com/steve.williams7/ A major revision of the Edinburgh Handedness Inventory.pdf). Results showed that nine participants were right-handed (mean laterality quotient: 87, *SD* = 14.2), and one participant was mixed-handed (laterality quotient: −25). Participants reported having normal hearing and no neurological disorders, and gave informed written consent prior to the experiment. The study was approved by the local ethical committee of the Faculty of Behavioral Sciences at the University of Helsinki, and conducted in accordance with the Declaration of Helsinki.

### Stimuli

**Figure 1:**
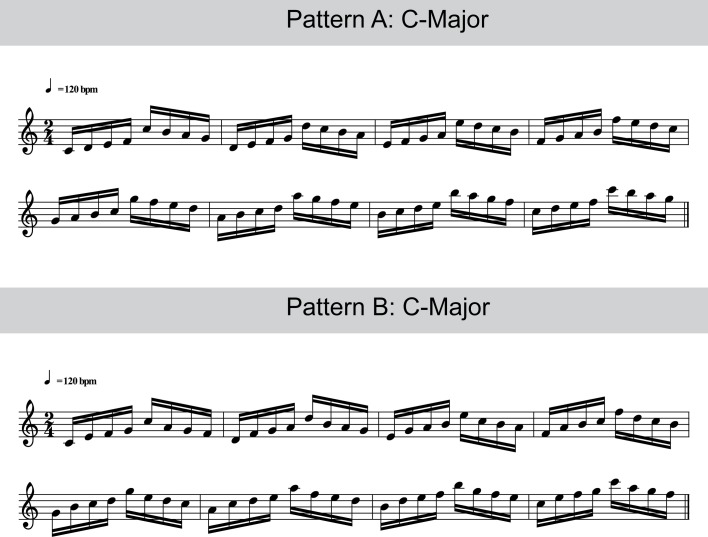
**Examples of the stimulus material.** Participants were required to perform such sequences with their right hand at an IOI of 125 ms, using the fingering 1-2-3-4-5-4-3-2-1-2-3-4-5-4… etc. (1, thumb; 2, index finger; 3, middle finger; 4, ring finger; 5, pinkie finger).

The stimuli consisted of two slightly different fingering patterns (see Figure 1). In each of the 56 experimental blocks, one type of pattern had to be produced with the right hand four times, in direct succession, in one of the following major keys: C-Major, D-Major, E-Major, F#-Major, G-Major, A-Major, or B-Major. The order of blocks was randomized with the constraints that no sequence in the identical key occurred in direct succession and that the same type of pattern was repeated a maximum of two times. Participants were instructed to use the same fingering throughout the experiment: 1-2-3-4-5-4-3-2-1-2-3… etc. (where 1 = thumb and 5 = pinkie). The instructed tempo for the sequences was 120 beats per minute—or 2 beats per second—for quarter notes. Because the required 16th notes have a duration of one quarter of a quarter note, this tempo resulted in an IOI between notes of 125 ms.

The sequences were performed on a Yamaha digital piano (S90XS; Yamaha Corporation, Japan), and participants listened to their performances with AKG 240 studio headphones at comfortable listening levels (dependent on the velocities of the key depressions). Due to technical difficulties, 4 participants performed on a Yamaha KX88 digital piano. Importantly, results of post-experimental questionnaires showed comparable levels of playing comfort between the two pianos. All tones had a standard MIDI piano timbre, generated by a Roland XV-2020 (Roland Corporation, Japan) synthesizer module.

### Procedure

Participants received the musical scores and tempo instructions prior to the experiment and were asked to memorize and rehearse the sequences (without looking at their hands while playing) with their own instrument at home.

After EEG and motion capture preparations, participants sat in front of the piano in a light-dimmed room. Before the experiment, participants could familiarize themselves with the piano, warm up, and perform one practice block. Participants were instructed to play as accurately as possible in the given tempo, but they were unaware of the exact aim of the study. Before each block, an instruction appeared on the screen placed above the keyboard and informed the participant about the type and key of the pattern to be performed. Simultaneously, four metronome beats were played to remind them of the correct tempo. After that, a green fixation circle in the center of the screen signaled that the participant could start playing. After each block, there was a short break and participants were able to continue the experiment by pressing a button whenever they were ready. Participants wore a custom-made visor that prevented them from visually monitoring the keys and their hands while playing but still allowed looking straight ahead at the screen. At the end of the experiment, participants completed questionnaires about their musical backgrounds and about the experiment. The whole experiment, including breaks and preparations, lasted approximately 3–4 h and pianists were paid for their participation.

### Data recording

#### Musical data

MIDI data were recorded by a modified version of the FTAP software (Finney, 2001a,b). To synchronize MIDI, motion capture, and EEG data, the FTAP software sent synchronization signals concurrently with every fifth key press to the EEG recording device. Similarly, the motion capture system sent synchronization signals simultaneously with each recorded frame to the EEG recording device (for details of this setup, see Maidhof et al., 2013).

#### EEG data

The EEG was recorded with a Biosemi ActiveTwo system (Biosemi, The Netherlands) from 64 Ag/AgCl active electrodes placed according to the extended 10–20 system (Fp1, AF7, AF3, F1, F3, F5, F7, FT7, FC5, FC3, FC1, C1, C3, C5, T7, TP7, CP5, CP3, CP1, P1, P3, P5, P7, P9, PO7, PO3, O1, Iz, Oz, POz, Pz, CPz, Fpz, Fp2, AF8, AF4, Afz, Fz, F2, F4, F6, F8, FT8, FC6, FC4, FC2, FCz, Cz, C2, C4, C6, T8, TP8, CP6, CP4, CP2, P2, P4, P6, P8, P10, PO8, PO4, and O2). The horizontal electrooculogram (EOGH) was recorded from electrodes placed at the left and right outer canthi, and vertical EOG (EOGV) was recorded from electrodes placed above and below the left eye. Two additional electrodes were placed at the left and right mastoid. EEG signals were digitized with a sampling frequency of 8192 Hz. Low-pass filtering during recording was performed digitally in the ADC's decimation filter, which has a 5th-order sinc response with a -3 dB point at 1638.4 Hz (see also http://www.biosemi.com/faq/adjust_filter.htm).

#### Motion data

Eight infrared Qualisys ProReflex cameras (Qualisys, Sweden) recorded the three-dimensional position data of 25 reflective markers (4 mm in diameter) with a sampling frequency of 120 Hz. The markers were attached to the fingernails, each finger joint, the wrist, and the back of the right hand of each participant. Additional markers were placed on the C4 and B4 keys. Only the data from the five markers at the fingertips are reported here.

### Data analysis

#### Musical data

Performance errors were detected offline by using the MIDI toolbox for MATLAB (Eerola and Toiviainen, 2004) and its extension for matching a musical performance to its corresponding notation (Large, 1993). After identifying errors, only pitch (or substitution) errors were further analyzed; such substitution errors occur when participants play a different note than written in the score. Other error types like note omissions and additional notes were discarded. Furthermore, pitch errors entered the analysis only if they were preceded by at least three correct notes, and correct notes entered the analysis only if they were preceded and followed by at least three correct notes. IOIs of correct and incorrect notes were calculated by subtracting the MIDI onset of the previous note from the MIDI onset of the current note—that is, by calculating *n* minus *n*−1 (IOIs thus refer to pre-note intervals). Only notes (correct and incorrect) that showed IOIs between 50 and 300 ms, and whose duration was between 50 and 180 ms, were selected.

Next, we calculated the mean MIDI keystroke velocity and mean IOI of correct and incorrect notes for each participant. Then, a subset of correct notes of each participant was created that included only correct notes that showed the same IOI as the mean IOI of incorrect notes (±5 ms). This selection of notes allowed for a comparison of correct and incorrect notes played with approximately the same tempo.

#### Motion data

The interaction between the finger and a rigid body (i.e., a key) during a keystroke can be determined by two kinematic landmarks (see Goebl and Palmer, 2008, 2009): a key-bottom contact (KB) landmark occurs when the finger's motion ceases as the key reaches the key bed, that is, when the key is fully pressed down; and a finger-key contact (FK) can occur when the finger initially touches the surface of the key before any pressure to the key is applied, that is, when the finger makes first contact before the key is actually pressed down (key depression). Both landmarks involve an abrupt change in acceleration and are therefore characterized by a peak in acceleration in the height-dimension of the finger's trajectory. Similar to Goebl and Palmer (2008), an FK landmark was identified when an acceleration peak in the finger trajectory was larger than 20 m/s^2^ in a time window ranging from −150 to −20 ms (i.e., prior to the MIDI onset). KB landmarks were identified when the acceleration peak in the finger trajectory was larger than 5 m/s^2^ in a time window ranging from −10 to 35 ms around MIDI onsets. Note that the kinematic data were analyzed independently from the EEG data.

#### EEG data

EEG data were processed offline in MATLAB (7.10.0) using the freely available toolbox EEGLAB 10.2.5.8b (Delorme and Makeig, 2004) and custom routines. To reduce the data size, EEG data were down-sampled to 256 Hz. Data were filtered by applying a high-pass filter [0.5 Hz, 3508 points, finite impulse response filter (FIR)], and subsequently a low-pass filter (45 Hz, 164 points, FIR).

Before performing an Independent Component Analysis (ICA), data segments contaminated with untypical and gross-movement artifacts were visually identified and removed. The cleaned datasets were then subjected to an extended Infomax version of ICA as implemented in the runica algorithm in EEGLAB. The resulting independent components (ICs) were screened for artifactual components due to eye blinks and movements, and electrode artifacts. An IC was considered to represent activity from eye blinks and movements if its topography showed peak activity only over the horizontal or vertical eye electrodes, if it showed a smoothly decreasing power spectrum, and if the component's activity contributed primarily to the raw EEG signal recorded by horizontal and vertical eye EOG. Identified artifactual ICs were subtracted from the data, which were subsequently low-pass filtered (25 Hz, 36 points, FIR), and re-referenced to the arithmetical mean of both mastoid electrodes.

Epochs representing single experimental trials time-locked to the MIDI and FK onsets of correct and incorrect keystrokes were extracted from −400 to 600 ms, respectively. To investigate if differences between the ERPs of correct and incorrect notes reflect differences in processing the previous notes rather than error detection mechanisms, two random subsets from the pool of all correct notes were compared. Each subset comprised approximately three times the number of incorrect trials for each participant. This procedure also reduced the amount of correct notes. Epochs were then baseline corrected (from −400 to −200 ms), and subjected to an automatic artifact rejection procedure, which discarded trials that showed larger or smaller amplitude values than +60 μV or −60 μV, respectively.

Consequently, ERPs time-locked to the MIDI onset were, on average across 10 participants, computed for (a) 105 incorrect keystrokes (±46); (b) a first subset of 300 correct keystrokes (±151); (c) a second subset of 298 correct keystrokes (±154); (d) 387 correct but slow keystrokes (±335); and (e) 105 correct pre-error notes (±48). ERPs time-locked to the onset of FK landmarks were, on average across all participants, computed for (a) 75 incorrect keystrokes (±41); (b) a first subset of 217 correct keystrokes (±116); (c) a second subset of 216 correct notes (±114); and (d) 78 correct pre-error notes (±41). For the comparisons of the two subsets of correct notes and correct pre-error notes, one participant had to be excluded because too few trials could be distributed into the two subsets. Similarly, the number of correct but slow notes was too small for this participant (*n* = 21). Note that the number of trials used for the ERP computation differs depending on whether the ERPs are time-locked to the MIDI onset or to the onset of the FK landmarks. This is because not all keystrokes showed a clear FK landmark (see Results section), and because the artifact rejection procedure resulted in discarding different trials.

#### Statistical evaluation

Based on visual inspection of the grand-averaged scalp topographies, mean ERP amplitude values were initially calculated for three regions of interest (ROIs): one frontal ROI (including electrodes F3, FZ, and F4), one central ROI (electrodes C3, CZ, and C4), and one parietal ROI (electrodes P3, PZ, P4). Time windows for statistical analysis were chosen based on the visual inspection of the grand-average ERPs and centered around the maximum of the differences between two conditions. Behavioral and movement data were statistically analyzed using paired sample *t*-tests. ERP data were analyzed with repeated measures ANOVAs with the factors Keystroke (correct, incorrect) and Frontality (frontal, central, parietal). The reported *p*-values were corrected using the Huynh-Feldt method where appropriate. The significance level for all tests was 0.05. All statistical analyses were performed with the software package PASW Statistics 18. ERPs were low-pass filtered (20 Hz) for presentation purposes only.

## Results

### Behavioral results

#### Tempo

The mean IOI between two correct successive keystrokes was 129.4 ms (*SD* = 31.3 ms), indicating that participants were able to perform in the instructed tempo of 125 ms. Incorrect keystrokes were performed with a prolonged IOI (*M* = 143.8 ms, *SD* = 31.4 ms) compared to correct keystrokes [*t*_(1, 9)_ = −6.27, *p* < 0.0001]. In addition, the IOIs of correctly performed notes immediately preceding erroneous keystrokes (“pre-error” notes) were prolonged (*M* = 137.9 ms, *SD* = 30.2 ms), compared to IOIs of correct keystrokes [*t*_(1, 9)_ = −1.31, *p* = 0.025].

#### MIDI velocity

The MIDI velocities of incorrect keystrokes (*M* = 61.2, *SD* = 7.1) were decreased compared to correct keystrokes [*M* = 68.4, *SD* = 7.2; *t*_(1, 9)_ = 5.61, *p* < 0.0001]. However, MIDI velocities of pre-error notes (*M* = 70.2, *SD* = 4.7) and correct notes did not differ [*t*_(1, 9)_ = −1.49, *p* = 0.17].

#### Kinematic results

**Figure 2:**
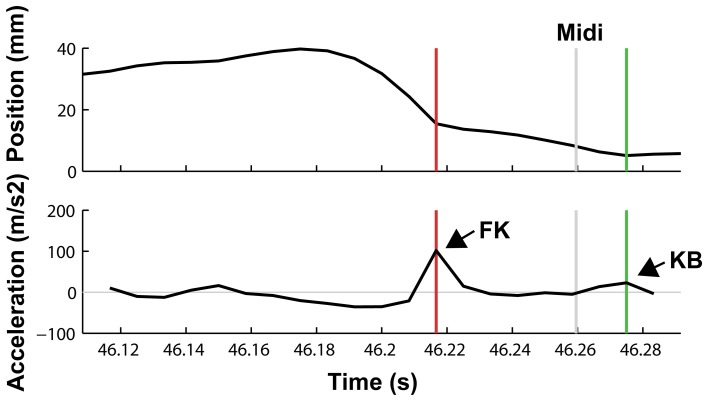
**Vertical motion of the fingertip of a pianist's ring finger playing the G#5 key.** Upper panel: finger position, lower panel: acceleration. An acceleration peak can be observed around 40 ms prior to the MIDI note onset, occurring when the fingertip makes initial contact with the key surface (finger-key landmark, FK). Shortly after the MIDI onset, another acceleration peak occurs when the key reaches the key bed after key depression (key-bottom landmark, KB).

An example of a keystroke showing a finger-key landmark (occurring when the finger touches the surface of the piano key) followed by a key-bottom landmark (occurring when the key reaches the key bed) is shown in Figure 2.

Results revealed that the majority of keystrokes showed a key-bottom landmark. In 99.8% of all correct and 97.7% of all incorrect keystrokes, a KB landmark was detected. However, the number of KB landmarks differed significantly between the two conditions [*t*_(1, 9)_ = 4.99, *p* = 0.001]. In contrast, percentages of keystrokes with an FK landmark did not differ between correct and incorrect keystrokes (ca. 78% for correct keystrokes and ca. 82% of all incorrect keystrokes; *p* = 0.44). KB landmarks occurred around 13 ms after MIDI onset, regardless of the correctness of keystrokes (*p* = 0.62). However, the mean amplitude of KB landmarks of incorrect keystrokes (27.9 m/s^2^, *SD* = 3.2 m/s^2^ was decreased compared to correct keystrokes [34.3 m/s^2^, *SD* = 4.6 m/s^2^; *t*_(1, 9)_ = 6.21, *p* < 0.0001]. This is consistent with the decreased MIDI velocity and indicates that erroneous keystrokes were performed with slower downward movements than correct keystrokes.

In contrast, FK landmarks during incorrect keystrokes occurred significantly earlier than FK landmarks during correct keystrokes. On average, FK landmarks of correct notes occurred 51.4 ms (*SD* = 8.3 ms) prior to MIDI onsets, whereas FK landmarks of error notes occurred 59.6 ms (*SD* = 8.3 ms) prior to MIDI onsets [*t*_(1, 9)_ = 3.4, *p* = 0.008]. The distances of the FK landmarks with respect to the MIDI onset of the previous note did not differ between correct and incorrect keystrokes [*t*_(1, 9)_ = −1.207, *p* = 0.258], and occurred on average around 80 ms after the MIDI onsets. The mean amplitude of FK landmarks was around 48 ms/s^2^ and did not differ between correct and incorrect keystrokes (*p* = 0.27).

Although correct notes immediately preceding incorrect notes showed a prolonged IOI, they did not differ from other correct keystrokes in terms of percentage, amplitude, or latency of KB and FK landmarks (*p*'s > 0.2).

### ERP results

#### MIDI-based

**Figure 3:**
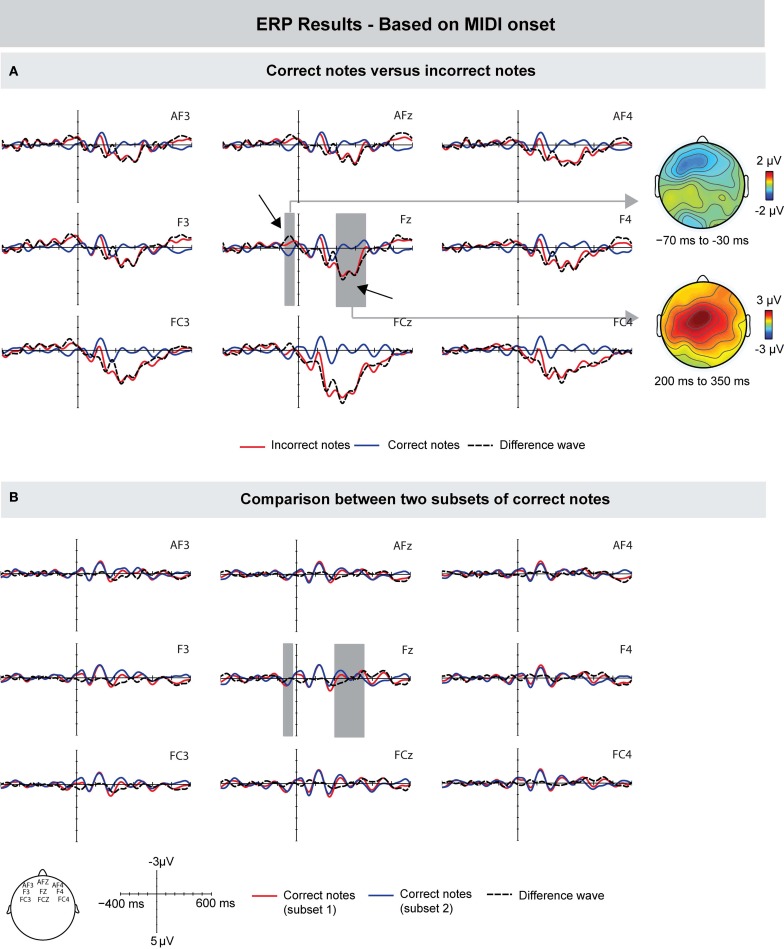
**Grand-averaged ERPs (left) and scalp topographies (right) based on the MIDI data. (A)** Compared to correctly produced keystrokes, incorrectly produced keystrokes elicited a pre-error negativity around 50 ms prior to MIDI onset, and an Error Positivity (Pe) around 250 ms after MIDI onset. **(B)** To investigate whether the difference between correct and incorrect notes is influenced by overlapping ERP responses from previous notes, two subsets of correct notes were compared. However, ERPs did not differ in the time windows used for the comparison between correct and incorrect notes. Gray areas show the time windows used for statistical analysis, exemplarily on one electrode. On the right side, corresponding scalp topographies are shown as the difference potentials between two conditions in the given time windows.

First, we compared the ERPs time-locked to the onset of the MIDI signal of incorrect and correct keystrokes, similar to previous studies (Herrojo Ruiz et al., 2009; Maidhof et al., 2009; Herrojo Ruiz et al., 2011). Compared to correct keystrokes, incorrect keystrokes showed an increased negativity prior to the onset of a keystroke. The difference was maximal around 50 ms prior to the onset of the key press, and showed a frontally distributed scalp topography (see Figure 3A). An ANOVA for a time window of −70 to −30 ms showed an interaction between factors Keystroke and Frontality [*F*_(1, 9)_ = 4.741, *p* = 0.048], and no main effects (*p*'s > 0.14). A follow-up analysis showed an effect of Keystroke only over the frontal ROI [*F*_(1, 9)_ = 7.147, *p* = 0.025]. This early difference was followed by a positive deflection showing maximal amplitudes around 250 ms after the MIDI onset over more central leads. An ANOVA for a time window of 200–350 ms showed main effects of Keystroke [*F*_(1, 9)_ = 13.632, *p* = 0.005], Frontality [*F*_(1, 9)_ = 5.005, *p* = 0.019], and an interaction between these factors [*F*_(1, 9)_ = 5.226, *p* = 0.016], indicating that amplitude values were larger over central leads.

To investigate whether the observed ERP differences were influenced by overlapping ERP responses from previous notes (e.g., change detection in pitch) and thus to investigate whether the above-mentioned findings are error-specific, we compared the ERPs of two random subsets of correct notes. The rationale was that if ERP effects reflect mainly error-related processes, the comparison of random correct notes should not show any differences. The results of this comparison are shown in Figure 3B. ANOVAs in the same time windows of the pre-error negativity and the later positivity (i.e., from −70 to −30 ms, and from 200 to 350 ms, respectively) showed no differences between the ERPs of randomly selected subsets of correct notes (all *p*'s > 0.32).

**Figure 4:**
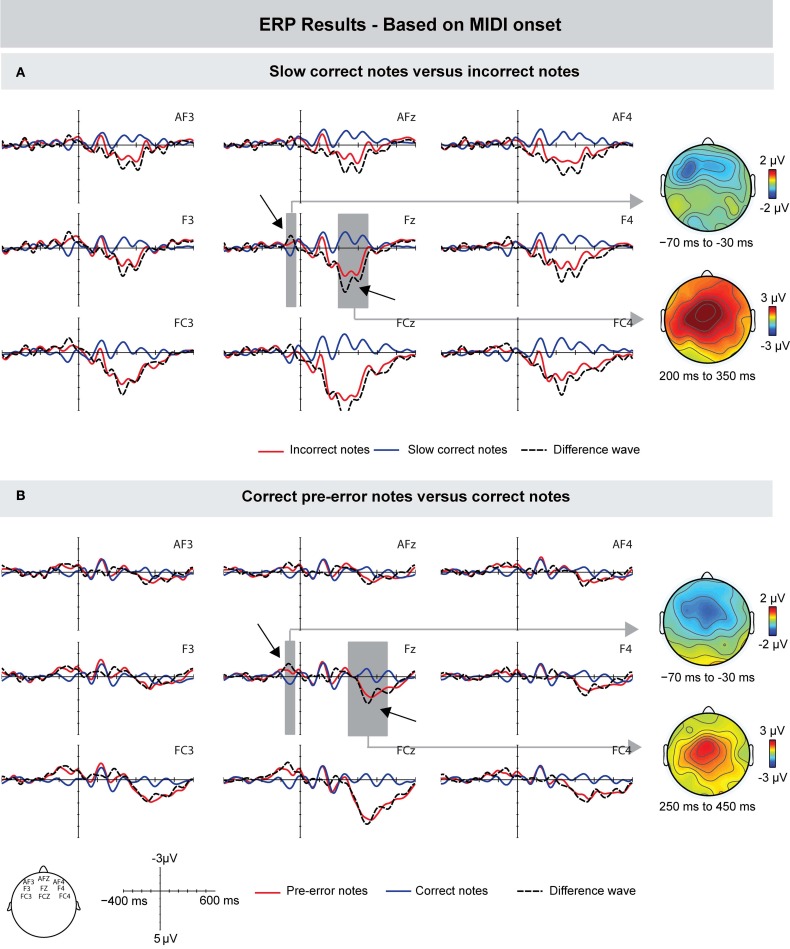
**Grand-averaged ERPs (left) and scalp topographies (right) based on the MIDI data. (A)** Compared to correct (but slow) keystrokes, incorrect keystrokes elicited a similar (marginally significant) negativity peaking around 50 ms prior to MIDI onset, followed by a Pe around 220 ms after MIDI onset. **(B)** Compared to other correct keystrokes, the correct keystrokes immediately preceding incorrect keystrokes elicited a negativity that preceded MIDI note onsets by about 60 ms, followed by a positivity peaking around 350 ms after MIDI note onsets. Gray areas show the time windows used for statistical analysis, exemplarily on one electrode. On the right side, corresponding scalp topographies are shown as the difference potentials between two conditions in the given time windows.

Next, we investigated the influence of different IOIs of correct and incorrect keystrokes on the ERP effects. Figure 4A shows the grand-averaged waveforms time-locked to the onset of the MIDI signal of incorrect keystrokes and a subset of correct keystrokes: only correct keystrokes that matched the mean IOIs of incorrect keystrokes ±5 ms were analyzed (see Method section for details). In line with previous results, incorrect keystrokes elicited an increased negativity prior to the onset of a keystroke compared to (slow, but) correct keystrokes. The difference was maximal around 60 ms prior to the onset of the MIDI signal, and showed a frontal scalp distribution. However, this difference was only marginally significant: an ANOVA for a time window ranging from −70 to −30 ms showed a marginally significant interaction between factors Keystroke and Frontality [*F*_(1, 9)_ = 4.248, *p* = 0.058]. Subsequent ANOVAs computed for the three ROIs separately showed only an effect that was approaching significance over the frontal ROI [*F*_(1, 9)_ = 3.473, *p* = 0.095].

The early difference was followed by a positive deflection peaking around 220 ms after key press onset with maximal amplitudes over more central leads. An ANOVA for a time window of 200–350 ms showed main effects of Keystroke [*F*_(1, 9)_ = 33.277, *p* < 0.0001], Frontality [*F*_(1, 9)_ = 4.398, *p* = 0.028], and an interaction between these factors [*F*_(1, 9)_ = 6.458, *p* = 0.008], indicating that this effect was more centrally distributed.

Furthermore, we compared the ERPs elicited during correct events that immediately preceded incorrect pitch events with the ERPs elicited during other correct pitch events (see Figure 4B). Results showed that (correct) pre-error notes also elicited, compared with other correct notes, an increased negativity prior to the MIDI onset. This difference peaked around −60 ms and showed a more central scalp topography. An ANOVA for the time window of −70 to −30 ms showed a marginally significant effect of Keystroke [*F*_(1, 9)_ = 4.852, *p* = 0.055], and an interaction between the factors Keystroke and Frontality [*F*_(1, 9)_ = 4.955, *p* = 0.025]. The early difference was followed by a positivity with a peak latency of around 350 ms (starting around 300 ms): an ANOVA in a time window of 250–450 ms showed a marginally significant effect of Keystroke [*F*_(1, 9)_ = 4.721, *p* = 0.058]. Given the mean IOI of ~130 ms, the latency of 350 ms is consistent with the latency of the positivity elicited by incorrect notes (around 220 ms). Hence, the late positivity seen in the ERPs of pre-error notes is most likely due to the following incorrectly produced note.

#### Motion-based

**Figure 5:**
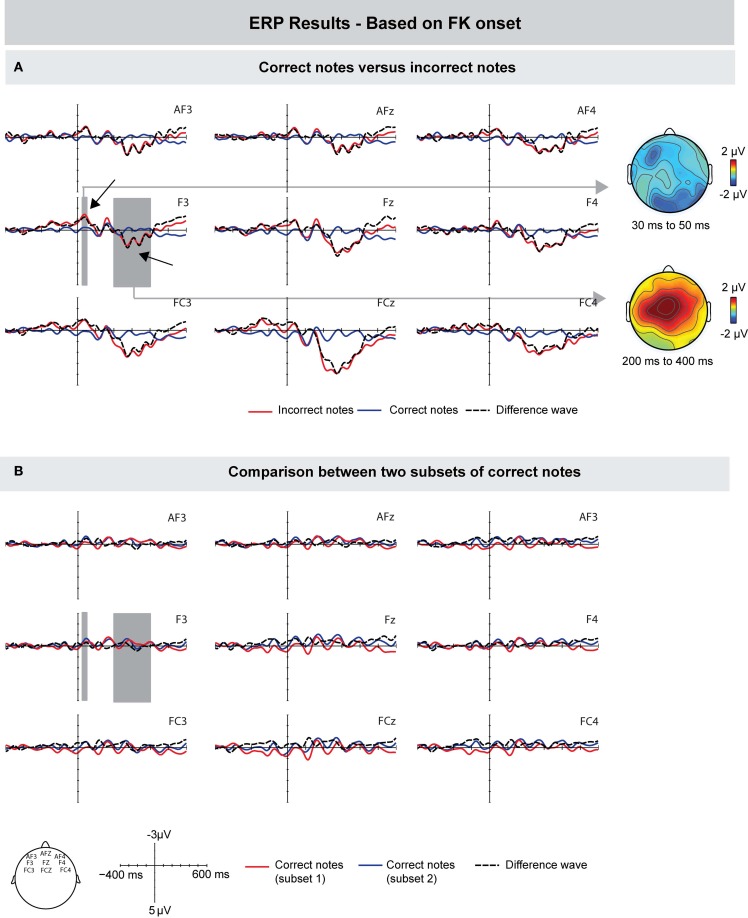
**Grand-averaged ERPs (left) and scalp topographies (right) based on the movement data.** ERPs are time-locked to the finger-key landmarks (FK) in the motion trajectories, occurring when a finger makes initial contact with the surface of a key. **(A)** Compared to correctly produced keystrokes, incorrectly produced keystrokes showed a marginally significant increased negativity around 40 ms after FK onset, which was focused only to left-frontal electrodes. Incorrect notes elicited a positive deflection peaking around 280 ms after the onset of tactile feedback. **(B)** To investigate whether the difference between correct and incorrect notes is influenced by overlapping ERP responses from previous notes, two subsets of correct notes were compared. ERPs showed marginally significant differences in the time windows used for the comparison of correct and incorrect notes. Therefore, results of the ERPs time-locked to FK onsets can only be tentatively interpreted. Gray areas show the time windows used for statistical analysis, exemplarily on one electrode.

To investigate the role of tactile feedback during erroneous keystrokes, we computed the ERPs relative to the onset of FK landmarks, that is, when a finger touches the surface of a key before it is pressed down (instead of relative to the MIDI onset, which occurs when the key is already pressed down). Figure 5A shows that incorrect pitch events, compared to correct pitch events, elicited a small negative deflection with a peak latency around 40 ms after the onset of tactile feedback. However, this difference seemed to be focused only to left-frontal electrodes F3 and FC3. An ANOVA for a time window of 30–50 ms after FK landmark onset with the same ROIs used for the other analyses showed no effect of Keystroke and no interaction with this factor (*p*'s > 0.19). Upon close visual inspection of the grand-average waveform, we conducted an additional ANOVA for the means of electrodes F3 and FC3, which showed a marginally significant effect of Keystroke [*F*_(1, 9)_ = 4.469 *p* = 0.059]. Importantly, there was no difference prior to the onset of tactile feedback (ANOVA for the same time window as in the MIDI-based ERPs, i.e., −70 to −30 ms: *p*'s > 0.18). Around 280 ms, incorrect notes elicited a positive deflection with a central scalp distribution. An ANOVA for a time window of 200–400 ms over frontal, central, and parietal ROIs showed main effects of Keystroke [*F*_(1, 9)_ = 7.752, *p* = 0.021], Frontality [*F*_(1, 9)_ = 4.382, *p* = 0.03], and an interaction between Keystroke and Frontality [*F*_(1, 9)_ = 4.578, *p* = 0.025]. Note that the latency of this positivity is consistent with the peak latency of around 220 ms after MIDI onset, given that FK landmarks occurred around 50 ms prior to MIDI onsets.

To investigate whether this small difference was influenced by overlapping ERP responses from previous notes, we again compared the ERPs of two random subsets of correct notes (analogous to the comparison for the ERPs based on the MIDI signal; Figure 5B). Results showed that ERPs of only correct notes differed marginally significantly in the same time window as the difference between correct and incorrect keystrokes was observed [ANOVA for a time window of 30–50 ms over electrodes F3 and FC3: *F*_(1, 8)_ = 4.932, *p* = 0.057]. Thus, it seems that overlapping ERP responses from previous notes influenced the difference between correct and incorrect notes, and therefore that results of ERPs time-locked to the onset of FK landmarks can only be interpreted tentatively.

**Figure 6:**
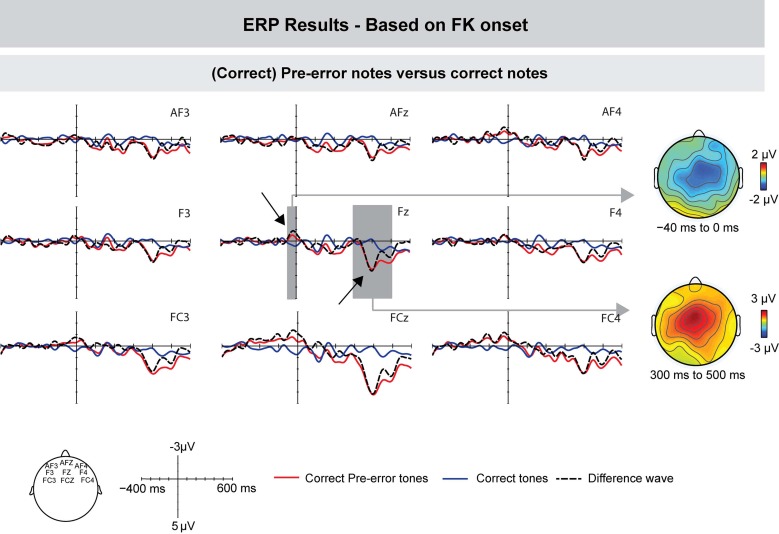
**Grand-averaged ERPs (left) and scalp topographies (right) based on the movement data.** Compared to other correct keystrokes, pre-error notes showed an increased negativity shortly before FK onset, followed by a positive deflection around 350 ms. Gray areas show the time windows used for statistical analysis, exemplarily on one electrode. On the right side, corresponding scalp topographies are shown as the difference potentials between two conditions in the given time windows.

The ERPs of correct notes immediately preceding incorrect notes and ERPs of other correct notes are depicted in Figure 6 (for this analysis, one participant was excluded due to there being too few trials). In contrast to correctly produced pitch events (elsewhere in the sequences), pre-error notes elicited no negative deflection shortly after the onset of tactile feedback (ANOVA for a time window of 0–50 ms: *F*'s < 1). However, pre-error notes showed an increased negativity prior to the onset of tactile feedback: an ANOVA for a time window of −70 to −30 ms showed a marginally significant effect of Keystroke [*F*_(1, 8)_ = 3.851, *p* = 0.085] and an interaction between Keystroke and Frontality [*F*_(1, 8)_ = 4.88, *p* = 0.028], indicating that the effect was more pronounced over central leads.

Around 350 ms after FK onset, pre-error notes elicited an increased positive deflection, compared to other correct notes; an ANOVA for a time window of 250–450 ms showed a marginally significant effect of Keystroke [*F*_(1, 8)_ = 4.721, *p* = 0.058].

## Discussion

The present study investigated errors during the performance of musical sequences by using a novel setup combining the recording of EEG, MIDI, and 3D movement data (for details of this setup, see Maidhof et al., 2013; for a non-musical setup aiming at mobile EEG recording of freely moving participants, see Makeig et al., 2009). This allowed us to investigate the underlying movements of pitch errors during piano performances, but also to exploratively relate the neurophysiological findings to different movement phases.

Replicating previous behavioral findings (Herrojo Ruiz et al., 2009; Maidhof et al., 2009; Herrojo Ruiz et al., 2011; Strübing et al., 2012), erroneous keystrokes were executed with reduced intensities (in terms of MIDI velocities) compared to correct keystrokes. Furthermore, the IOIs of incorrectly produced pitch events were prolonged compared to correct keystrokes (by ca. 13 ms). However, the analyses of kinematic landmarks during keystrokes provided additional information about the underlying movements. Importantly, the onset of tactile feedback for incorrect pitch events (FK landmarks, occurring when a finger makes first contact with the key surface; Goebl and Palmer, 2008) occurred ca. 60 ms prior to key depression, whereas tactile feedback for correct pitch events started ca. 50 ms prior to key depression. Considering that incorrect events were produced ca. 13 ms slower than correct events, we assume that it was not the movement toward the next incorrect key itself that was executed slower, but rather that the phase between touching the key surface and complete key depression was prolonged for incorrect keystrokes. This assumption is supported by the finding that acceleration values did not differ when a finger made initial contact with the key surface during correct or incorrect pitch events. Thus, it is likely that the prolonged IOIs of incorrect notes are mainly due to the decreased velocity of those wrong keystrokes.

Interestingly, IOIs (but not MIDI velocities) of correctly produced notes immediately preceding incorrect notes were also prolonged, compared to other correct events elsewhere in a sequence. However, these pre-error notes did not differ in latency or amplitude of FK landmarks from other correct events. Thus, it remains unclear what exactly caused this prolongation, and future studies should investigate this effect in more detail.

ERPs based on the MIDI data replicated previous findings showing that incorrect keystrokes, compared to correct key presses, elicited an increased negativity already ~60 ms before a key was fully pressed down, and before auditory feedback of the error was available (pre-error negativity; Herrojo Ruiz et al., 2009; Maidhof et al., 2009; Strübing et al., 2012). This early negative potential was followed by a centrally distributed positive deflection, resembling the Error Positivity (Pe) or P300 component (note that Pe might actually reflect the same neural mechanisms as the P300; for reviews of the Pe, see Falkenstein et al., 2000; Overbeek et al., 2005).

However, these effects could be due to the different IOIs before correct and incorrect notes, and hence these findings could be confounded by the factor tempo. To exclude this possibility, we calculated the ERPs for a subset of correct notes that were closely matched to the tempo of incorrect notes. Results showed virtually the same ERP pattern, that is, an increased negativity prior to the MIDI onsets of incorrect notes compared to correct notes, and a subsequent P300 component.

Furthermore, these effects could also have been caused or influenced by overlapping ERPs (due to the short IOIs of around 125 ms) from other sensory-motor processes related to previous notes, irrespective of the correctness of the played notes. However, there are several reasons rendering it unlikely that the pre-error negativity and the Pe reflect simply overlapping sensory-motor processes and that they are not being elicited by pitch errors: first, the results of the comparison of two random subsets of correct notes showed no significant differences in the time windows of the pre-error negativity and the Pe, indicating that the effects are rather error-specific. Second, a previous study also reported that a pre-error negativity was also elicited in the complete absence of auditory feedback (Herrojo Ruiz et al., 2009), suggesting that the processing of previous pitch information did not significantly contribute to this effect. Third, a symbolic resonance analysis aimed at disentangling overlapping ERPs during piano performance validated the previous ERP results (Herrojo Ruiz et al., 2009).

In sum, these results, in combination with the kinematic data, suggest that the pre-error negativity and the Pe during piano errors are unlikely only due to tempo differences between correctly and incorrectly produced pitch events, and unlikely only due to overlapping ERPs elicited by previous notes. Instead, it appears that the early negative potential and the following positivity reflect error-related processes.

To investigate the role of tactile feedback for error-related processes during piano performance, we exploratively computed ERPs based on the onset of tactile feedback for each keystroke, which was determined based on the movement data. Results suggest that incorrectly produced pitch events did not differ prior to the tactile feedback of keystrokes, but that they were associated with a slightly increased negativity peaking around 40 ms after tactile feedback was available.

However, these findings can only be interpreted tentatively, because the comparison between the ERPs of two random subsets of correct notes revealed a difference in the time window of 0–50 ms. This indicates that, when ERPs were time-locked to the onset of tactile feedback, the difference between correct and incorrect keystrokes might have been influenced by overlapping ERP responses elicited by previous notes. In addition, the motion capture system had a relatively large sampling interval of 8.3 ms, and therefore the detection of FK landmarks was not as accurate as in previous studies (Goebl and Palmer, 2008; Palmer et al., 2009). Therefore, averaging across FK landmarks might have “smeared” the ERPs considerably, which could also have contributed to the difference between correct notes. It would be interesting to see whether the same results would be obtained with a motion capture system with a higher sample rate.

Nevertheless, one might speculate about the current finding of a negative difference potential after the onset of tactile feedback. One possible interpretation is that tactile and proprioceptive feedback may play an important role in the detection of upcoming performance errors: it is conceivable that, based on the tactile feedback of the key surface (but not earlier, for instance during the movement toward the key), the monitoring system can compare the predicted with the actual sensory consequences of a movement in the form of reafferent tactile information. By contrast, when the auditory feedback of keystrokes or of speech acts is externally manipulated, the earliest information with which the predicted consequences can be compared is the auditory feedback. Thus, brain responses after the onset of the auditory feedback are increased (or inhibitory effects are canceled) when a mismatch is detected (e.g., Houde et al., 2002; Heinks-Maldonado et al., 2006; Eliades and Wang, 2008; Katahira et al., 2008; Maidhof et al., 2013, 2010; Behroozmand and Larson, 2011; Behroozmand et al., 2011). However, when participants commit errors, information available even earlier, including tactile feedback, could be used for error detection. In this regard, predictions regarding the consequences of movements would include predictions of incoming tactile as well as auditory information, which is consistent with the notion that efference copies can interact at several stages of sensory processing (Crapse and Sommer, 2008). Furthermore, corrective modulations of the ongoing motor act can be initiated whenever a mismatch is detected. These corrective modulations can include the slowing of the ongoing keystroke resulting in a decreased loudness of the incorrect pitch, as indexed by the decreased MIDI velocity (and the corresponding lower acceleration values of finger trajectories during incorrect pitch events).

Note that the negativity peaking shortly after the touch of a key could also be interpreted as an error-related negativity in the context of existing theories of action monitoring. For example, the mismatch detection hypothesis holds that the ERN is elicited when a comparator detects a mismatch between the correct response and the actual response, and subsequently triggers an error signal (Falkenstein et al., 2000; Coles et al., 2001). Similarly, the Reinforcement-Learning theory of the ERN (for a review, see Nieuwenhuis et al., 2004), posits that the earliest available information about incorrect performance will generate an error signal. In the case of piano performance, one can speculate that the tactile feedback from the finger arriving at a key (and the proprioceptive feedback about the position of the finger) could represent the first indication of an incorrect performance.

However, from the present data it is difficult to conclude whether the negative difference reflects the error signal itself, or the initiation of behavioral adjustments to prevent the error (or at least to decrease the sensory effects caused by the error, i.e., reduce the loudness).

When time-locked to MIDI onsets, (correct) pre-error notes elicited a similar ERP pattern as incorrect notes, although the early negativity showed a slightly more central scalp topography. However, when time-locked to tactile feedback, the tentative ERP results of pre-error notes did not differ from other correct notes after the onset of tactile feedback, but only shortly before tactile information was available. Therefore, one might speculate that different and/or additional neural processes are operating during the execution of correct notes preceding wrong pitch events. These results might indicate that the monitoring system detected some problem in motor execution or planning (possibly resulting in an increased IOI), which, however, had not yet resulted in an incorrect pitch event. Hence, there is no mismatch between predicted and actual tactile feedback, and no correction has to be initiated, and thus no negativity is elicited after tactile feedback (of the correct key). Clearly, more research is needed to investigate this issue in more detail.

Taken together, the present study provided some further insights into the neural mechanisms of error processing during music performance, by combining electrophysiological with detailed three-dimensional movement data in an exploratory approach. Furthermore, the results tentatively suggest that the tactile feedback of piano keys plays a major role for predictive error processes, although future studies are needed to validate this interpretation. Interestingly, correct notes preceding errors showed similar neural activity to pitch errors themselves, although only pitch errors elicited increased neural activity after the onset of tactile feedback. In the future, we believe that the combined acquisition of electrophysiological and movement data can lead to a more behaviorally-driven analysis of brain activity during the performance of music. This approach also offers interesting possibilities in terms of conducting studies in other domains such as in music learning and education, music therapy, action-perception interactions, and musical expressivity in cross-sectional and—importantly—longitudinal paradigms, to reveal the time course and sensitivity of processes involved in learning and therapy.

### Conflict of interest statement

The authors declare that the research was conducted in the absence of any commercial or financial relationships that could be construed as a potential conflict of interest.
